# Beyond the Bench: Oasis of Fun at Mount Desert Island

**DOI:** 10.1289/ehp.113-a376

**Published:** 2005-06

**Authors:** Tanya Tillett

Kids, summer, and water are three things that just naturally go together. The Community Outreach and Education Program (COEP) of the Center for Membrane Toxicity Studies, housed at Mount Desert Island Biological Laboratory in Bar Harbor, Maine, is using its open-air Myers Marine Aquarium Visitors Center to maximize this combination and get kids excited about learning during the summer months. The Center for Membrane Toxicity Studies, one of the NIEHS Marine and Freshwater Biomedical Sciences Centers, educates children about the environment and their own role in keeping it healthy.

For about five years, the COEP has provided environmental health enrichment to the surrounding community through the Myers Marine Aquarium Visitors Center. Aquarium staff provide weekly programs in the summer for local and tourist visitors to learn about the center’s research. In a typical tour, visitors learn about water pollutants and how they affect our health and environment, and are also given information on water quality management.

Because school-aged children are the target audience, the message delivery is kept simple and fun. The aquarium has several tanks that allow for viewing and touching of the lab’s research species and other sea life from the Gulf of Maine. Cartoon-emblazoned posters illustrate center findings on how pollutants are processed in the body and describe in nonspecialist language our own responsibility in safeguarding the environment we live in. As part of the tour, center staff describe their research projects and allow visitors to view specimens through microscopes.

“When we talk about environmental stewardship, I am always amazed by the reaction of the children,” says Jeri Bowers, the COEP associate director. “They love to get involved and to feel like they are playing an important role in maintaining a healthy environment; they take it very seriously. Our hope is that we can capture that enthusiasm and interest while they’re young—and influence their behavior well into adulthood.”

The aquarium attracts large numbers of student groups. One particularly memorable visit was a May 2004 tour by a group of 40 children with Almström syndrome, a rare hereditary disorder that can affect multiple organ systems and cause blindness, hearing impairment, type 2 diabetes mellitus, heart failure, and liver disease. The visit was arranged by researchers at the nearby Jackson Laboratory, who discovered the gene for Almström syndrome and provide their own outreach to children affected by the illness. Bowers says the tactile component of the aquarium experience was especially important and beneficial for these children, most of whom can’t see or hear.

Along with the strong community outreach it offers through the aquarium and labs, Mount Desert Island also has exciting environmental health research in progress that staff hope will someday become part of the children’s experience. Earlier this year, the lab set up a website (http://ctd.mdibl.org/) for the Comparative Toxicogenomics Database, a data system being created to provide centralized data on diverse organisms to scientists worldwide. The lab has also received NIH approval to sequence the genome of the skate, data that will eventually be entered into the Comparative Toxico-genomics Database. One of the future goals of the researchers is to incorporate the electronic data into the visitors program so that children can access the database on computers set up as part of the aquarium tour.

## Figures and Tables

**Figure:**
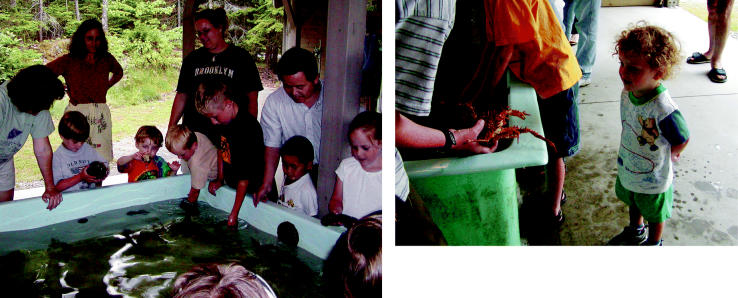
**Diving in.** Children learn about the marine environment and their role in keeping it healthy at the Myers Marine Aquarium Visitors Center.

